# Implementation of diabetic foot ulcer classification system for research purposes to predict lower extremity amputation

**DOI:** 10.4103/0973-3930.50707

**Published:** 2009

**Authors:** AbuBakr H. Widatalla, Seif EIDin I. Mahadi, Mohamed A. Shawer, Hagir A. Elsayem, Mohamed E. Ahmed

**Affiliations:** Jabir Abu Eliz Diabetic Center, University of Khartoum. Khartoum, Sudan

**Keywords:** Amputation, diabetes, diabetic foot, ischemia, neuropathy

## Abstract

**BACKROUND:**

Patients with diabetic foot ulcers are at a high risk of having both minor or major lower extremity amputations.

**AIM:**

To identify the extent of risk factors for major and minor amputations in patients with diabetic foot ulcers.

**MATERIALS AND METHODS:**

This prospective study was conducted from 2003 to 2005. Using the guidelines for wound classification developed by the International Consensus of the Diabetic Foot, patients were assessed for ischemia, neuropathy, linear measurement of wound diameters, depth of wound, and infection. In addition, end stage renal failure was added as a criterion to assess the association of all these criteria with both toe and lower extremity amputation.

**RESULTS:**

2,321 patients were studied and their mean age was 55 ± 12 years. Most (83.5%) of the patients presented with foot ulcers (*n* = 1394). Plantar ulcers were the most common (42.6%) followed by ulcers of the big toe (39%). Some (28.5%) of the patients had different types of amputations: 10% had major lower extreme amputation (MLEA) with 8.7% amputations being below the knee and minor (toe) amputations accounting for 18.5%. The most commonly amputated (9.9%) toe was the first toe.

**CONCLUSION:**

The guidelines for wound classification proposed by the International Consensus of the Diabetic Foot are reliable predictive factors and can determine the outcome of diabetic foot management. Significant factors associated with MLEA were ischemia, neuropathy, and end-stage renal disease and those associated with toe amputation were neuropathy, depth of wound, and grade of infection.

## Introduction

Diabetic patients face 15–40 times greater risk of amputation over the course of their lives than do nondiabetic individuals.[1] In Sudan, foot complications of diabetes are common, costly, and disabling. Sound understanding of the pathogenesis, the spectrum and treatment of the disease is poor among health professionals. Major lower extremity amputations continue at the rate of 30–40% for inpatients in Khartoum's main teaching hospital.[2]

The establishment of a Diabetic Foot Centre in Khartoum in 1998 triggered a huge influx of patients with an average of 80 patients, including 6–8 new cases, coming daily for wound care. Several risk factors that predict the outcome of the diabetic wound have been identified by various authors.[3–5] However, very few studies on diabetic foot management and outcome have been reported from the African continent.[6] The objective of this study was to use the wound classification criteria adopted by the International Consensus of the Diabetic Foot[7] to predict the outcome with regards to minor and major lower extremity amputation. Based on the findings of a recent, local study, renal failure was added as a co-morbidity.[8]

## Materials and Methods

A prospective study was conducted in JADC in Khartoum from 2003 until 2005 and included all patients presenting to the Diabetic Foot Clinic during this period. Data were collected using a predesigned data collection sheet after getting informed consent from all patients and analyzed using the SPSS system, version 12.

All patients were assessed for ischemia, neuropathy, depth of wound, infection, linear measures of the wound surface, and renal function impairment. The following criteria were applied for each index:

### A) Perfusion

Perfusion was considered as:

Grade 1: If the patient had no symptoms or signs of ischemia, the dorsalis paedis and posterior tibial pulses were palpable, and the ankle brachial index was between 0.9 and 1.1.

Grade 2: If the patient had symptoms and signs not of critical limb ischemia but of intermittent claudication, or the ankle / brachial index was < 0.9 but with ankle pressure > 50 mm Hg

Grade 3: Critical limb ischemia as defined by systolic ankle pressure < 50 mm Hg

### B) Depth of wound

Grade 1: Superficial ulcers not penetrating any structure below the dermis

Grade 2: Deep ulcers penetrating down to subcutaneous structures, fascia, muscles, and tendons

Grade 3: Deep ulcers penetrating down to the bone and /or joint

### C) Infection

Grade 1: No symptoms or signs of infection

Grade 2: Infection involving skin and subcutaneous tissues only without systemic signs

- Local swelling and induration
- Erythema > 0.5–2 cm around ulcer
- Local tenderness or pain
- Local warmth
- Purulent discharge

Grade 3: - Erythema > 2 cm

- Deep abscess, osteomyelitis, septic arthritis, and fascitis

Grade 4: any foot infection associated with systemic inflammatory response syndrome

- Temperature > 38 or < 36°C
- Heart rate > 90 beats/min
- Respiratory rate > 20 breaths/min
- TWBCs > 12,000 or < 4000/cm

### Sensation

Grade 1: No loss of sensation of the affected foot

Grade 2: No pressure sensation with a 10 g monofilament on two or three sites on the plantar side of the foot. No vibration sense with a 128 Hz tuning fork on both sides of the hallux.

## Results

2,321 diabetic foot patients were seen at JADC during the period 2003–2005. The mean age (± SD) for the study population was 55.5 ± 12.3 years; the majority of patients (71%) had type 2 diabetes mellitus. Many (83.5%) patients presented with foot ulcers and blisters were seen in 55.0% of the patients with an offensive smell detectable from the wound in 15.9% of the patients. Edema of the affected limb was found in 36.3%, and tender visible veins indicating thrombophlebitis in 6.7% of the patients. A few (10%) patients had fever at presentation and 25% of them had a history of general weakness and prostration. Examination of the wounds revealed tissue necrosis in 39.0%, gangrene in 12.5%, and pus discharge or collection in 46.4% of the patients.

No inflicting cause was identified in the majority of the patients (40.4%). Sharp injuries were reported in 17.8%, new shoes in 13.0%, thermal injuries in 4.5%, and various causes in 24.3% of the patients.

The most commonly affected toe was the big toe in 39.0% of the patients, followed by the second toe in 18.5% of the patients. The third, fourth, and fifth toes were affected in 10.7, 7.3, and 9.8% of the patients respectively.

The plantar aspect of the foot was affected in 42.6% of the patients whereas only the heel was involved in 10% of the patients. Wounds were found on the heads of the metatarsal bones in 9.7% of the patients. The lateral and medial aspects of the foot and dorsum of the foot were involved in 7.2, 12.8, and 13.6% of the patients respectively.

Some (28.5%) patients had different types of amputations: 10% had MLEAs with amputations below the knee accounting for 8.7% while the rest had amputations above the knee. The first toe was the most commonly amputated in 9.9% of the patients.

Forefoot and Syme's amputations were carried in 3.0 and 1.3% of the patients respectively.

A few (10.5%) patients had critical limb ischemia (perfusion grade 3)—this was found to be the most significant risk factor for major amputations (P = 0.000, odds ratio = 5.08; CI = 2.56–10.07).

Grade 1 sensory neuropathy was found in 42.6% of the patients and grade 2 in 57.4%. Peripheral sensory neuropathy was found to be significantly associated with MLEA (P = 0.027, odds ratio = 2.43; CI = 1.08–5.45) and with minor toe amputation (P = 0.002, odds ratio = 2.16; CI = 1.32–3.5). Grade 2 sensory neuropathy was found to be associated with deep ulcers penetrating down to the bone or joints in 21% of the patients whereas Grade 1 deep ulcers occurred only in 13.7% (*P* = 0.001).

Superficial ulcers occurred in 41.7% of the patients, ulcers penetrated down to the subcutaneous fascia and muscles in 42.2%, and to the bones or joints in 16.0% of the patients. Deep ulcers penetrating down to the subcutaneous fascia, muscles, tendons, bones, and joints were found to be significantly associated with toe amputation (P < 0.005, odds ratio = 3.45; CI = 2.02–5.88).

Infection was diagnosed in 63.6% of the patients (grade 1 in 36.4%, grade 2 in 33.0%, grade 3 in 26.6%, and grade 4 in 4.0% of the patients)..Grades 3 and 4 infection were found to be significantly associated with toe amputation (*P* < 0.005, odds ratio = 2.4; CI = 1.55–3.7).

Patients with end-stage renal failure represented 3.2% of study population. End-stage renal disease (patients on dialysis) was found to be significantly associated with MLEA (*P* = 0.003, odds ratio = 4.39; CI = 1.53–12.61).

**Figure 1:**
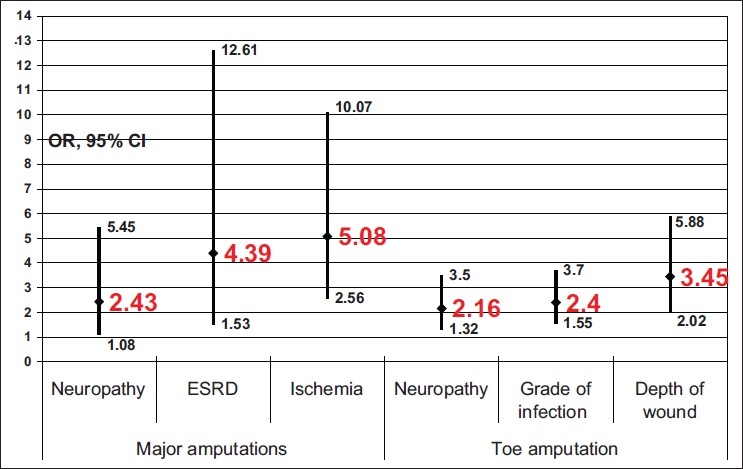
Factors significantly associated with amputation

Both wound diameters and the surface area were not found to be significantly associated with any type of amputation. A summary of the factors linked with both major and minor amputations is shown in Figure 1. Significant association of MLEA was found with major limb ischemia and ESRD whereas minor toe amputation was significantly associated with deep wounds and those with grades 3 and 4 sepsis. Neuropathy was associated with both minor and major amputations.

## Discussion

Foot ulcer is the main presentation of a diabetic foot in our patients (85%) in nearly half of whom it was preceded by a blister. The unsatisfactory initial management of the blister by patients and health professionals was the main factor leading to a long protracted course that tended to end in an amputation. A major concern for both patients and treating physicians when faced with a diabetic foot is to forecast an outcome based on the available data on presentation. We used the criteria for wound classification adopted by the International Consensus for the Diabetic Foot to get a reliable grading of the diabetic foot and predict the outcome. These criteria were: the degree of limb ischemia, sensory neuropathy, depth and surface area of the wound, severity of sepsis, and ESRF. Our data are consistent with the findings of many previous studies that have reported that peripheral vascular disease in diabetic patients is an independent risk factor for lower limb amputation,[9–15] In the absence of vascular intervention in our setup, peripheral vascular disease was the most significant indication for MLEA (*P* = 0.000). Similar data have been reported from other developing countries due to a lack of revascularization service.[16] The integrated care of critically ischemic diabetic patients by diabetologists, vascular surgeons, and podiatrists offers them a beneficial major / minor amputation ratio.[17–19]

The significant association between peripheral neuropathy in diabetic patients and lower limb amputation has been documented in many previous reports.[10–13,15] Absence of sensation with the 10 g monofilament and with 128 Hz vibration was associated with MLEA as well as toe amputation in patients presenting with deeply infected neuropathic ulcers with osteomyelitis. Deep and severe neuroapthic ulcers have been found to be significantly associated with severe neuropathy. Major amputations were also recommended for patients with advanced Charcot's arthropathy with severe destruction of the bones and ankle joints.

Several systems of ulcer classification have been used in the literature, the most popular one was found to be Wagner's classification based on six grades of ulcer depth.[20] However, this system omitted two important factors: the degree of infection and the extent of ischemia. The University of Texas San Antonio (UTSA) system added both infection and ischemia to the ulcer dimension and hence, became more widely used.[21]

Many diabetics with end-stage renal disease suffer from advanced progression of the disease and suffer from an extremely high risk of lower limb amputation.[14,22] The coincidence of peripheral vascular disease and peripheral neuropathy is far more common in these patients than in patients without renal impairment. Coexistence of peripheral vascular disease and medial sclerosis is far more common in the dialysis group.[23] In our study population, we found that major amputation rates are significantly higher in patients with end-stage renal disease (*P* = 0.003).

With respect to the depth of the wounds, lower-grade lesions responded well to conservative treatment with surgical debridement and antibiotics whereas those with higher grades needed amputations.[24,25] In our study population, we found that the deeper wounds were significantly associated with toe amputation (*P* < 0.05), which could be attributed to the local damage of tissues, necrosis, and osteomyelitis of the phalanges and the metatarsal bones.

Infection was a complicating factor in 50% of our patients who presented with foot ulcer (85%) and systemic infection in the form of fever or prostration was reported in 35% of them. When infection complicates a diabetic foot, the combination can be limb- or life-threatening.[13,26,27] The major predisposing factors to these infections were foot ulceration and various immunological disturbances.[26] Aerobic gram-positive cocci, especially *Staphylococcus aureus,* have been found to be the predominant pathogens in diabetic foot infections.[25,26] In our study population, we reported infection in 63.6% of the patients with *Staphylococcus aureus* being the most common isolate (33.2%). Patients with deep sepsis and osteomyelitis (Grade 3) had significantly higher rates of toe amputations. The combination of osteomyelitis and deep-seated sepsis has been found to be associated with 62% of amputations compared with 37 and 30% for each component independently.[28] This is due to deep tissue necrosis and local ischemia of the toes because of septic arteritis and thrombosis of digital arteries.

The guidelines developed by the International Consensus are helpful in understanding the pathophysiology of the diabetic foot and planning of its treatment. Also, the guidelines are reliable predictive factors that determine the outcome of the management. To reduce the amputation rate, however, attention should be paid through a multidisciplinary team to timely referral from the physician, patient education, total contact cast, and appropriate revascularization.[29,30] The situation is more challenging in developing countries due to limited resources so that more stress should be given to prevention, patient education, and the establishment of multidisciplinary teams in small diabetic units that disseminate and apply the international guidelines on the management of the diabetic foot.
